# Effectiveness of an empowerment-based self-defense program among South African girls: results from a cluster-randomized control trial in schools

**DOI:** 10.1186/s12905-025-03647-w

**Published:** 2025-03-14

**Authors:** Miriam Hartmann, Shepherd Mutangabende, Stephen Nash, Erica N. Browne, Abigail Hatcher, Anna E. Kågesten, Sarah T. Roberts

**Affiliations:** 1 Women’s Global Health Imperative, RTI International, Berkeley, CA USA; 2 Department of Global Public Health, Karolinska Institutet, Stockholm, Sweden; 3 No Means No South Africa, Gqeberha, South Africa; 4 Department of Medical Epidemiology and Biostatistics, Karolinska Institutet, Stockholm, Sweden; 5 University of North Carolina, Chapel Hill, NC USA

**Keywords:** [list 3–10] adolescent girls, Sexual violence, Cluster-randomized control trial, South Africa, Schools, Empowerment self-defense

## Abstract

Empowerment-based self-defense (ESD) programs have proven effective in preventing sexual violence (SV) among girls in diverse settings, yet their effectiveness in South Africa remains unexplored. In this hybrid type 1 cluster-randomized controlled trial, we assessed the impact and implementation of the COVID-adapted ‘No Means No’ intervention, an ESD program to prevent SV among girls aged 10–19 in Gqeberha, South Africa. Fifteen schools were randomly assigned to one of three trial arms: an arm receiving intervention delivered to girls-only, one receiving interventions delivered to girls and boys seperately, or to serve as controls in a 1:1:1 ratio. Surveys were conducted at baseline, 3, 6, and 12 months. The primary outcome was past-year SV exposure among girls, including harassment or rape. Generlized Estimating Equation models compared the two intervention arms to the control, and each intervention arm to the control. Implementation data consisted of intervention attendence logs, quarterly implementation reports, and in-depth interviews with school stakeholders. We enrolled 1,540 from 14 schools, of whom 1,250 provided primary outcome data. The average age was 13 years and 83% identified as Black. At baseline, 35% of girls reported past-year SV, and 33% did during follow-up. The intervention did not significantly reduce SV compared to the control (adjRR 1.24, 95% CI 0.96, 1.69; *p* = 0.08). Exploratory analyses examined the effects on secondary outcomes (rape, offline, and online sexual harassment), and on intermediate outcomes (e.g., knowledge and attitudes) with only knowledge of self-defense differing between intervention and control (coefficient 0.42, 95% CI 0.16, 0.68; *p* = 0.007). Implementation data revealed barriers to implementing in schools post-COVID, including limitations on alloted time and lack of whole-school buy-in. Improved school awareness, willingness to disclose violence, and reductions in bullying were described as impacts of the intervention by stakeholders. While the lack of reduction in SV may point towards areas for improvement, numerous contextual and implementation factors may also have influenced results. Future trials should utilize implementation science methods to improve delivery and rigorously evaluate ESD interventions’ impact on disclosure, acknowledging the complexity of assessing their effects on various facets of SV. The trial was registered on clinicaltrials.gov (NCT05295342).

**Supplementary Information:**

The online version contains supplementary material available at 10.1186/s12905-025-03647-w.

## Introduction

Sexual violence (SV) is a significant public health issue, with global implications for achieving Sustainable Development Goals 3 (good health and well-being) and 5 (gender equality) [1, 2]. The elimination of all forms of violence against women and girls is crucial not only for creating a more equal society but also for improving population physical and mental health. In South Africa, the situation is particularly dire. One prospective cohort study among adolesents, age 10–19, found that 23.9% of girls reported exposure to SV, with exposure increasing with age (7% among 10–14 year olds vs. 31.7% among those 15 and up) [3]. Other studies have found that up to 65% of South African women have experienced violence at the hands of their partners [4], and non-partner sexual violence rates are also alarmingly high, with estimates ranging from 6 to 25% of women experiencing rape [5, 6].

Given the pervasive nature of SV, there is an urgent need for effective interventions. Research indicates that early prevention, particularly through school-based interventions, holds promise [7]. School-based programs can target younger adolescents, addressing norms around violence and fostering skills in recognizing, avoiding, and reporting sexual violence [8, 9]. Although much of the evidence comes from high-income countries, there are promising examples from low- and middle-income countries (LMICs) as well [10]. One such example is an empowerment-based self-defense (ESD) intervention delivered in schools in Kenya, which was found to significantly reduce sexual assault [11].

ESD is an intervention approach grounded in feminist theories that seek to counteract power imbalances faced by women in gender-inequitable societies. ESD trains women and girls in verbal and physical techniques to recognize, prevent, and stop SV. This approach frames SV as a structural issue, aiming to counteract norms that blame women and girls for violence while empowering them with skills to respond [12–14]. A recent review of self-defense programs for SV prevention found strong evidence of their effect in reducing nonconsenual– forced or coerced– sex among girls, adolescents, and adult women in a variety of settings worldwide [15]. However, none have been tested in South Africa, where widespread SV occurs alongside many other intersecting forms of violence [16]. To further explore the effectiveness of ESD, this hybrid type 1 cluster-randomized controlled trial evaluated a COVID-adapted ESD curriculum delivered in primary and secondary schools in South Africa by the organization No Means No (NMN) - South Africa.

The primary aim of this study was to test the effectiveness of the intervention on girls’ self-reported SV, including forced sex and online and offline sexual harassment. Building on existing evidence, we also aimed to understand its impact on intermediary outcomes theorized to be mechanisms of change, such as victim-blaming attitudes and confidence in reporting SV, as well as to identify contextual barriers and facilitators associated with implementation at the school-level.

## Methods

### Overall study design

The *No Means No (NMN)* evaluation study was a hybrid type 1 effectiveness-implementation design [17] using a three-arm, parallel, cluster-randomized control trial for superiority conducted among 15 co-ed schools in Gqeberha, South Africa. Primary and secondary schools were included since NMN delivers their intervention to both school levels. Schools were chosen as the randomisation unit to avoid contamination given that the intervention was classroom-based. The selected schools were randomized to receive the NMN girls intervention only, seperately delivered girls and boys interventions, or standard of care (control condition) in a 1:1:1 ratio. Here we present the effect of the intervention on girls; results from the boys’ outcomes will be presented elsewhere. The primary comparison is between the two intervention arms combined and the control arm. We used a list of all schools in the Gqeberha school district as our sampling frame and eliminated schools that were outside of the central city. We then stratified by school type (primary vs. secondary) and language (Afrikaans vs. isiXhosa) and selected five sets of three schools from the same strata, based on location to ensure geographical separation to reduce potential contamination and for feasibility. Randomization occurred after all schools were matched into sets stratified by school type and language of instruction (see Table 1). Schools were randomized within sets. Following randomization one intervention arm school from the isiXhosa-speaking secondary school set chose not to participate and a back-up school from the stratum was used to replace it [18]. Additonally, one control-arm school from the Afrikaans-speaking primary school set refused to participate at a stage where it was too late to replace them, and a second intervention-arm school from the Afrikaans-speaking secondary school set stopped participation after baseline data collection, but prior to intervention implementation. This resulted in a sample of 13 schools. Due to these losses, two out of the five sets were incomplete and so we here we present results based upon an unmatched analysis. The trial was registered on clinicaltrials.gov (NCT05295342) in March 2022.

**Table 1:** Characteristics of matched sets

Matched set	School type	Language	Notes
1	Primary	Afrikaans	Control school dropped out after randomization
2	Secondary	Afrikaans	School allocated to Girls only intervention arm dropped out after baseline
3	Primary	isiXhosa	
4	Primary	isiXhosa	
5	Secondary	isiXhosa	School allocated to Girls and Boys intervention arm dropped out shortly after randomization and was replaced

### Study setting and context

The study took place in Gqeberha, formerly Port Elizabeth, the largest city in the Eastern Cape province of South Africa and part of the Nelson Mandela Bay Metropolitan area. The municipality accounts for 18% of the total population in Eastern Cape province. It has the second highest overall crime index of the sub-regions within the overall Eastern Cape province, with a high number of sexual crimes reported. A total of 23,376 sexual offences were reported in the province in 2019/2020, a category that includes rape, compelled rape, sexual assault, incest, bestiality, statutory rape and the sexual grooming of children [16]. Unemployment is high, and a high proportion of its population live in poverty (50.70% in 2016) defined as not being able to purchase both sufficient food and non-food items [19]. Gqeberha is also the site of a NMN innovation and learning ‘Hub’, the only site worldwide where NMN delivers programs directly rather than through partners. It was launched in 2020, during the time of COVID, when South Africa faced numerous waves of infections and went through various stages of social restrictions that intermittendely closed schools– the site of intervention implemention– as well as exacerbated several known drivers of gender-based violence, such as mental health, unemployment, and food insecurity [20, 21].

### Sample size and power

The planned sample size was 120 girls per school in each of 15 schools (1800 total; 600 per arm). This would have given 80% power to detect a minimum of 5–7% reduction in the incidence of SV between the combined intervention arms and the control arm, assuming an intracluster correlation coefficient (ICC) of 0.0025–0.005 and SV incidence of 15-20% per year in the control arm. Re-estimating the power based on the actual enrolled sample size and observed SV incidence in the control arm, we had 80% power to detect 8.2-9.0% reduction in the incidence of SV.

### Study population, recruitment, and data collection

Recruitment occurred from February-October 2022. Within each school, the research team worked with administrators to select 4–8 classrooms to receive the intervention or control condition and enroll in the study. Female participants were sensitized to the study at the classroom-level and verified for eligibility, including being aged 10–19, not planning to move schools during the study period, willing to participate in the intervention, and English, isiXhosa, or Afrikaans speaking. Participants signed either an informed consent if 18 years of age or older, or an assent alongside parental consent. Participants were followed for 12 months with surveys conducted at baseline, and months 3, 6, and 12. All surveys were self-administered on paper-based forms in the classroom, and data were double-entered into the secure web-based platform REDcap (Research Electronic Data Capture) [22]. NMN Instructors, who were not involved in delivering the intervention to the learners with whom they were collecting data from, read the survey questions out loud to the classroom to ensure comprehension regardless of reading level. Instructors were trained to emphasize honest reporting in order to inform intervention improvements and ensure that participant responses would not be shared with the intervention Instructors who taught them. Recruitment, enrollment, and data collection tools and methods were pilot tested in one primary and one secondary school prior to finalization.

A purposive sample of 10 school stakeholders (1 per intervention school), aged 18 and above and selected based on their roles coordinating with the intervention team, were invited to participate in an in-depth interview (IDI). Interviews were conducted by trained qualitative interviewers in a private location, in English, and were audio-recorded. Semi-structured guides were used to facilitate discussions on barriers and facilitators to implementation of the NMN intervention in the school setting with questions structured around the consolidated framework for implementation research (CFIR) [23], a globally recognized framework for understanding implementation issues within a research context. Audio-files were transcribed into English and underwent quality checks to ensure consistency with the audio-files.

### Intervention and control

The NMN COVID-adapted girls intervention consisted of sessions utilizing empowerment-based pedagogy and employing experiential learning methods like role playing to equip girls with practical tools for sexual assault situations. Session topics included understanding forms of violence, awareness of risks, verbal and physical skills such as verbal assertiveness and calling for help, as well as content designed to counter victim-blaming narratives. The NMN boys intervention focused on positive masculinity, gender equitable norms, and bystander intervention in violence. Both interventions were delivered in 8-hours, over 1–5 weeks in 1–2 h sessions during school hours and were conducted by trained NMN instructors. COVID adaptations included reducing the curricula delivery hours from 12 to 8-hours and for the girls curricula, content adaptations included emphasizing verbal skills over physical and adjusting the pedagogy associated with physical skill development, such that no physical contact occurred. The intervention followed a logic model developed by NMN, which proposed that the intervention content and approach would increase knowledge of self-defense, self-efficacy to utilize verbal and physical skills, and improve gender norms and victim-blaming attitudes among participants. These outcomes were theorized to increase SV disclosure and reduce SV. The intervention was delivered by instructors, who represent young people from the communities which they serve. Prior to implementation they underwent in-person training on the curricula methodology and were observed on a regular basis by a Program Manager to provide feedback for ongoing development and quality implementation. Standard of Care, received by the control arm, included Life Skills or Life Orientation classes, delivered by school staff. In all arms, certified social workers employed by NMN were tasked with following up on all reports of violence by participants and connecting participants to further care where indicated. A referral network offering psychosocial, medical, and legal support was available to all participants disclosing violence.

### Outcomes and measures

We conducted pre-testing of all questionnaire items with primary and secondary school learners to assess clarity, relevance, and cultural appropriateness, and ensure that the items were well-understood and applicable to the target population for accurate data collection.

### Primary outcome

The primary outcome, sexual violence, was defined *“as any sexual act*,* attempt to obtain a sexual act*,* unwanted sexual comments or advances*,* or acts otherwise directed against a person’s sexuality using coercion*,* by any person regardless of their relationship to the victim*,* in any setting”* [24]. Its presence was denoted by a postive response to any of the 15 items in a survey delivered at baseline, month 6, and 12. The baseline survey asked about experiences in the prior 12 months; the later two surveys asked about experience in the prior 6 months, which were combined to represent any exposure in the past 12-months. If the respondent did not answer any of the items the outcome variable was set to missing.

### Secondary outcomes

*Sexual harassment* was measured using 9-items. Five items measured offline harassment, which have been used in other evaluations of NMN [25] and were originally drawn from the American Association of University Women [26]. Four items measured online harassment [27], also known as technology-facilitated sexual violence. All items asked how often either a partner or someone else did specific behaviors to them (e.g. offline: touched, grabbed, or pinched you in a sexual way; online: publicly posted a naked or sexual photo of you). Each item had four response options for frequency: “never”, “a few times”, “once or twice a week”, or “every day or almost every day”. A binary variable was created where reports of any frequency of exposure more than “never” was categorized as experiencing that behavior. These were further combined into a binary variable indicating whether someone had experienced any sexual harassment in the past 12 months (0 = no, 1 = yes).

Experiences of rape were measured using 6-items. These included 4-items on forced or coerced sex from a partner or non-partner and 2-items on incapacitated sex, assessing whether someone had done something to the participant when they were too drunk or high to stop them, or gave them alcohol or drugs in order to do something sexual with them. Items have previously been used elsewhere for the evaluation of violence prevention interventions [28]. A binary variable was created to indicate whether someone had experienced rape in the past 12 months (0 = no, 1 = yes).

### Intermediary outcomes

Intermediary outcomes included outcomes theorized as contributing to change in SV according to the NMN logic model: *knowledge and attitudes towards self-defense*,* gender norm attitudes*,* victim-blaming attitudes*,* use of intervention behaviors* and *confidence in reporting SV*. All intermediate outcomes were measured at months 3, 6, and 12 except for knowledge and attitudes towards self defense, which was measured at month 3 only. Wherever possible, scales previously validated or used with adolescent girls were utilized.

*Knowedge and attitudes towards self-defense* and *use of intervention behaviors* were measured using questions adapted from prior evaluations of the NMN intervention in other settings [29]. The *knowledge* scale (range 11–29) was asked at month 3-only and included 11-items, with higher scores indicating greater knowledge. Five-items pertained to knowledge (e.g., “What is the main aim of self-defense?”) and 6-items measured attitudes around the right to defend oneself and use the skills taught, (e.g. “Is it okay to use force and even injure anyone who is known to me if he is forcing me to have sex and will not listen to me (e.g., brother, boyfriend, father, cousin)?”. Three questions, which were combined into a binary outcome (0 = no, 1 = yes), assessed whether someone had used verbal and/or physical skills to stop someone from forcing them to have sex, harassing them, or physically abusing them. The *gender norms and attitudes* scale consisted of 8-items, adapted for girls from the World Health Organization (WHO), with higher scores indicating more gender equitable attitudes (range 8–32) [30]. *Victim-blaming attitudes* consisted of 4-items, previously used in South Africa, with higher scores indicating less agreement victim-blaming attitudes (range 4–16) [31]. All scales were standardized using mean differences for ease of comparability and interpretation. *Confidence in reporting SV* consisted of a single likert scale item asking how much they agreed with the statement “if I were to experience SV, I would feel safe telling someone” (1 = totally disagree to 4 = totally agree). A binary variable representing any confidence in reporting was created (0 = no, 1 = yes) and a change in score from baseline to follow-up was calculated by subtracting the baseline response from follow-up (range: -3-3).

### Process evaluation measures

Process evaluation data included participant attendence, tracked by Instructors, as well as themes identified in quarterly implementation reports and barriers and facilitators identified via IDIs with school stakeholders.

### Analysis

#### Quantitative analysis

Descriptive statistics (e.g., frequencies, means) were used to summarize demographic characteristics of participants and attendence among intervention participants. Outcome analysis was performed using generalized estimating equations to account for clustering by school. We used an exchangeable correlation matrix, per recommendations [32], but conducted sensitivy analyses using independent and autoregressive correlation structures. A Poisson distribution and a log link function were utilized to calculate a risk ratio for SV comparing both intervention arms together to the control. The Fay and Graubard standard error correction was used due to high variability in cluster sizes [33] given that participants with follow-up outcome data per school ranged from 33 to 245, with a mean of 96.2 and a standard deviation of 58.3. The coefficient of variation of cluster sizes therefore was 58.3/96.2 = 0.61. In both models, we a priori adjusted for the randomization strata of school type (primary, secondary) and language (isiXhosa, Afrikaans), and the baseline value of the relevent outcome variable where possible [34]. We also looked at the effect of the intervention on experience of *rape* or *offline* or *online sexual harassment* separately and at the effect by intervention arm (i.e., the girls-only vs. control and girls and boys intervention arm vs. control). Finally, we analyzed the effect of the intervention on intermediary outcomes, i.e. *knowledge of self-defense*,* gender norm attitudes*,* victim blaming attitudes*, and *confidence reporting SV*, looking at the latest scores available for each participant. We used the same strategy for accounting for clustering as described above, but for continuous outcomes used a Normal distribution and an identity link in all models. The xtgeebcv command [35], which includes bias-corrected covariance estimates, in Stata v17 [36] (StataCorp 2021, College Station, TX) was used for all analyses with significance at the alpha = 0.05 level. Additionally, as further sensitivity analyses, we re-ran our model dropping all ‘small’ clusters, defined as those with less than 50 participants, and in line with reporting guidelines for cluster randomized control trials, ran a cluster-level analysis using a poisson regression model was conducted using the clan command [37].

#### Qualitative analysis

IDI transcripts were thematically coded and analyzed using Dedoose, a qualitative analysis software, by the first author. Coded data was extracted into Word documents for the development of analytical memos highlighting themes related to barriers and facilitators to school-based implementation according to the CFIR framework domains.

### Ethical considerations

The study included numerous ethical considerations given its inclusion of girls and boys below the age of 18 and the focus on violence. The study was reviewed and approved by the Human Sciences Research Council in South Africa (REC 2/17/03/21) and the Swedish Ethical Review Authority (Dnr 2022-03745-01). All participants provided written informed consent or assent (if under 18 years) prior to enrollment. Parental consent was sought for all minors. Procedures followed WHO ethical recommendations on conducting research on violence against women and girls [38, 39], such as rigorous training of the research team on ethical and safety guidelines for conducting such research, and followed a standardized protocol for responding to reports of SV using the LIVES (Listen, Inquire, Validate, Enhance Safety, Support) approach [40, 41] and reporting these cases according to the Children’s Act of 2005 in South Africa [42]. The study team also consisted of registered Social Workers, who responded to all reported cases of SV, regardless of their reporting mechanism (i.e. via survey responses or verbal disclosure).

## Results

### Participant characteristics at baseline

A total of 1,540 female participants were enrolled, of which 1,507 came from the 13 schools who completed the trial. The majority of girls were in secondary school (58%). Participants were on average 13 years old, majority Black (83%), and lived with an average of approximately 6 people in their household. Just over half reported ever having an intimate partner (52%), but only 7% reported having had sexual intercourse. Approximately one-third reported any experience of SV (35%). Participants reporting violence were on average 14 years of age, reported slightly higher food insecurity than participants as a whole (28.2% vs. 21%), were more likely to have reported having a boyfriend/girlfriend (80.2% vs. 52%), and primarily came from secondary schools (79.5%). See Table 2 for overall participant characteristics at baseline. Female participant characterstics by cluster are available as supplementary material (See Additional file 1, Table 1).

**Table 2:** Baseline characteristics of female participants in schools completing the trial, by study arm

	Girls’ Only Intervention Arm		Girls’ and Boys’ Intervention Arm		Control Arm		Total	
	*N*	%	*N*	%	*N*	%	*N*	%
Total	459	100	695	100	353	100	1507	100
Type of school								
Primary	243	(53)	205	(30)	179	(51)	627	(42)
Secondary	216	(47)	490	(71)	174	(49)	880	(58)
Age - *mean*,* median (IQR)*	12.6, 13	(11–14)	13.5, 14	(12–15)	13.1, 13	(11–15)	13.1, 13	(11–15)
Race/ethnicity								
Black	409	(89)	564	(81)	283	(80)	1256	(83)
Colored	36	(8)	104	(15)	58	(16)	198	(13)
White	3	(1)	5	(1)	5	(1)	13	(1)
Household size - mean, median (IQR)	6.7, 5	(4–8)	5.9, 5	(4–7)	6.4, 5	(4–7)	6.2, 5	(4–7)
Any food scarcity	87	(19)	147	(21)	80	(23)	314	(21)
Ever had a boyfriend/girlfriend	205	(48)	381	(59)	147	(44)	733	(52)
Ever had sex	30	(7)	65	(9)	10	(3)	105	(7)
Any SV outcome past 12 months (rape or offline/online sexual harassment)	124	(27)	300	(43)	104	(30)	528	(35)
Any rape past 12 months	54	(12)	113	(16)	38	(11)	205	(14)
Any offline sexual harassment past 12 months	88	(19)	250	(36)	83	(24)	421	(28)
Any online sexual harassment, past 12 months	60	(13)	158	(23)	54	(15)	272	(18)

Participant characteristics in all arms (columns) represent female participants. Rape was defined as forced, coerced sex, or sex while incapacitated due to drugs or alcohol. Sexual harassment included attempts to obtain a sexual act, unwanted sexual comments or advances, or acts otherwise directed against a person’s sexuality. These were either captured as ‘offline’ events or ‘online’ events that occurred via mobile apps, social networks, texts, or other digital communication. IQR = interquartile range. 12 selected “other” race and 28 did not respond. 107 were missing responses to household size, 169 to food scarcity, 47 refused to answer to history of having a boyfriend/girlfriend, 95 were missing history of sex, 89 were missing any SV, 85 to rape, and 90 to offline sexual harassment, and 89 to online sexual harassment questions

### Loss to follow-up

**Fig. 1:**
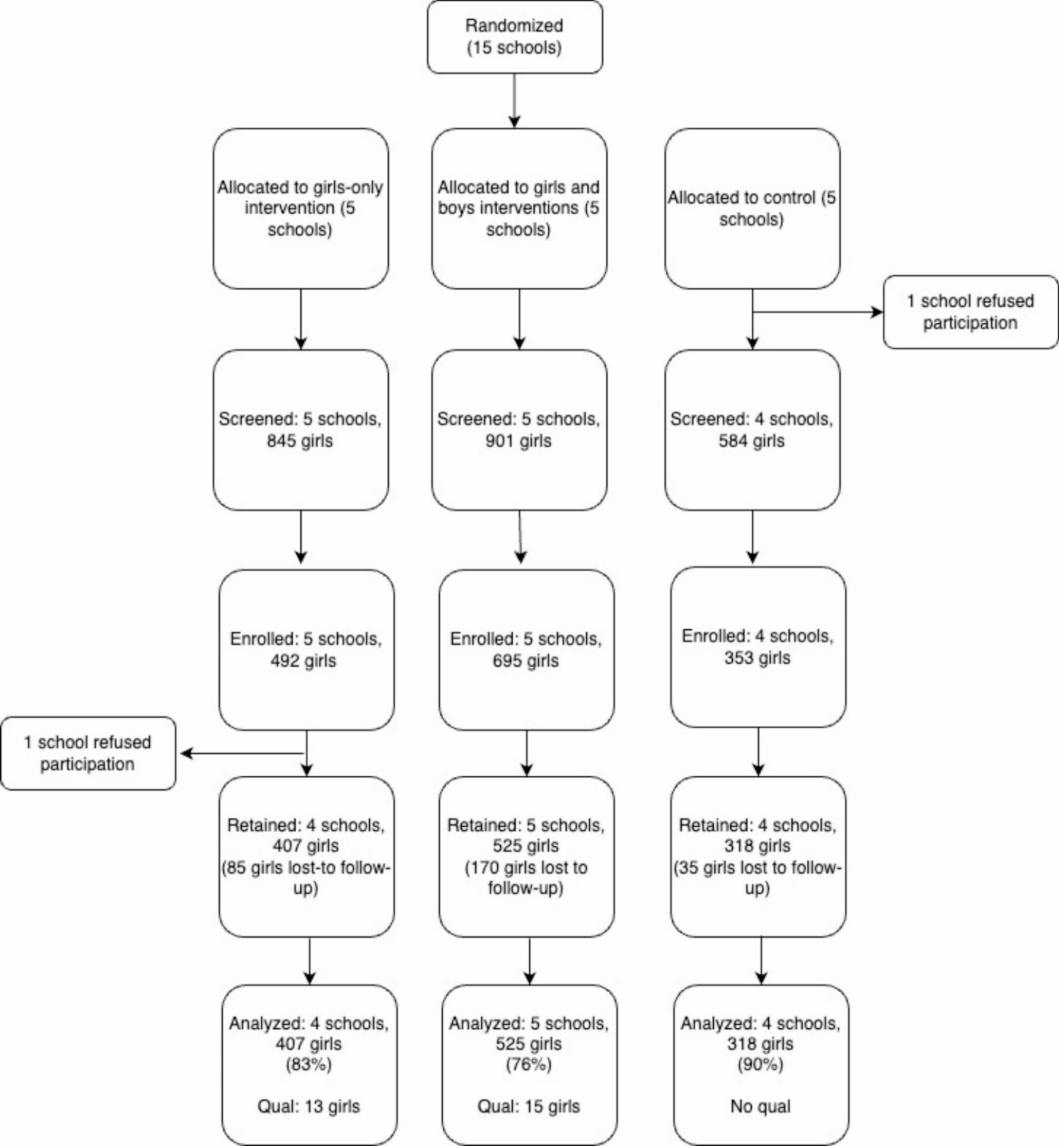
Study CONSORT chart of clusters and individuals

There were 1,250 participants from 13 schools with a primary outcome available out of the 1,507 enrolled. There was no relationship between reporting of SV during follow-up and amount of follow-up time available for a participant. However, follow-up time did differ by school level and by study arm. More primary school participants completed 12-months of follow-up than those in secondary schools (76% vs. 49%) and higher numbers of control arm participants completed the 12-months of follow-up, compared to the girls-only or girls and boys intervention arms (90% vs. 83% vs. 76%). Notably the average number of participants enrolled per control school was smaller than those in the intervention arms (88 vs. 126), which may account for this. See Fig. 1.

### Primary outcome

One-third of girls (33%;414/1250) reported SV during at least one visit over the 12-month follow-up period. Of thse, 52% had reported SV at baseline (215/414). Among girls in the intervention schools, 36% reported experiencing SV during follow up (336/932; Girls’ only: 140/407; Girls and boys’: 196/525) and 25% reported SV from among the girls in the control arm (78/318). Girls in the intervention arms had an estimated 24% higher risk of reported SV compared to girls in the control arm, although this was not statistically significant (adjRR 1.24, 95% CI 0.96, 1.69; *p* = 0.08). (see Tables 3 and 4a). Results from the analysis dropping small clusters (adjRR 1.20, 95% 0.96–1.51; *p* = 0.10) and the cluster-level analysis (adjRR 1.34, 95% CI 0.99, 1.79; *p* = 0.05) were similar to our main findings, indicating higher reporting of SV in the intervention arm (see Additional file 1, Tables 2 and 3).

When seperately comparing the two intervention arms to the control, participants in the girls’-only intervention arm had a 39% higher risk of reporting SV compared to the control arm (adjRR 1.39, 95% CI 1.06, 1.83; *p* = 0.03) whereas participants in the girls’ and boys’ intervention arm had a 17% higher risk (adjRR 1.17, 95% CI 0.89, 1.54; *p* = 0.23) compared to those in the control arm. See Table 4b. Results of the analysis looking separately at exposure to rape and sexual harassment followed the same trend as the primary analysis, and there were no statistically significant findings. See Additional file, Table 4.

**Table 3:** Sexual violence outcomes at follow-up among female participants by study arm

	Girls’ Only Intervention		Girls’ and Boys’ Intervention		Control		Total	
	*N*	%	*N*	%	*N*	%	*N*	%
Total	407		525		318		1250	
Any SV outcome past 12 months (rape or offline or online harassment)	140	(34)	196	(37)	78	(25)	414	(33)
Any rape past 12 months	65	(16)	69	(13)	32	(10)	166	(13)
Any offline sexual harassment past 12 months	99	(24)	157	(30)	58	(18)	314	(25)
Any online sexual harassment, past 12 months	78	(19)	83	(16)	37	(12)	198	(16)

**Table 4:** 4a and 4b. Generalized estimating equation model for primary outcome of sexual violence among female participants in the intervention arms combined and seperately, compared to control

		*N*	Adj RR	95% CI		*p*-value	
a	Intervention	932	1.24	0.96	1.69	0.08	
	Control	318	ref				
	Strata						
	High school, Afrikaans	145	0.89	0.62	1.28	0.45	
	Primary school, isiXhosa	536	0.56	0.44	0.71	0.001	
	Primary school, Afrikaans	70	1.04	0.74	1.47	0.80	
	High school, isiXhosa	499	ref				
	Baseline SV						
	Yes	386	2.34	1.82	2.99	< 0.001	
	Refused to answer	81	1.89	1.35	2.65	0.002	
	No	783	ref				
b	Girls’ only intervention	407	1.39	1.06	1.83	0.03	
	Girls’ and boys’ intervention	525	1.17	0.89	1.54	0.23	
	Control	318	ref				

Sexual violence (SV) defined as any sexual act, attempt to obtain a sexual act, unwanted sexual comments or advances, or acts otherwise directed against a person’s sexuality using coercion, by any person regardless of their relationship to the victim, in any setting. Adj RR = adjusted risk ratio; CI = confidence interval. Fay-Graubard bias-corrected standard errors. Model adjusted for baseline exposure to sexual violence, and strata defined by school type (primary, secondary) and language (isiXhosa, Afrikaans)

Participant characteristics in all arms (columns) represent female participants. SV = sexual violence. Rape was defined as forced, coerced sex, or sex while incapacitated due to drugs or alcohol. Sexual harassment included attempts to obtain a sexual act, unwanted sexual comments or advances, or acts otherwise directed against a person’s sexuality. These were either captured as ‘offline’ events or ‘online’ events that occurred via mobile apps, social networks, texts, or other digital communication.

### Intermediary outcomes

Results of our analysis of all other intermediary outcomes (i.e. *knowledge of self-defense*, g*ender norms*,* victim-blaming*,* use of intervention behaviors*, and *confidence in reporting SV*) indicated improved levels in the combined intervention arms compared to the control except for *gender norms*. However, only a difference in *knowledge of self-defense* at month 3 was statistically significant. Participants in the intervention arms had a knowledge score that was on average 0.42 units higher than the control arm (*p* = 0.007). When looking at the arms separately, significance held only for the girls’ and boys’ intervention arm (coefficient 0.56, 95% CI 0.35, 0.76; *p* = 0.001). The model for *use of intervention behaviors* did not converge due to small numbers of reporting use of intervention skills, all of which were reported at the month 6 visit with no reported behavior use at month 12 (Control arm: 2/318; Intervention arms: 20/932). Results are presented in Additional file 1 (see Additional File, Tables 5, 6 and 7).

### Process evaluation data

Among intervention arm participants, the majority (93%; range 84–100%) attended at least 6 out of 8 sessions. Quarterly implementation reports and stakeholders revealed numerous barriers and facilitators to implementation in schools following the COVID period. Themes with relevant quotes are presented in Table 5 by CFIR domain. Dominent themes at the *innovation domain* included positive views of the NMN staff, particularly a high value placed on the Social Workers and their efficacy as a neutral body to enhance disclosure. At the *outer setting domain* high levels of community violence were seen as barriers and facilitators in terms of the challenge it brought in terms of diverting school attention towards more immediate responses and acting as a motivating force. At the *inner setting domain* lack of school-wide engagement limited teacher buy-in and consequently limited time for implementation. However, school coordinators saw the intervention as a good fit and recalled numerous benefits felt at the school as a result, such as reduced bullying. Finally, at the *individual domain*, teachers themselves felt they learned skills from the ESD program, furthering their motivation and support.

**Table 4:** Barriers and facilitators to intervention implementation by CFIR domain

Barriers	Facilitators
***Innovation domain***	
Few stakeholders noted barriers specific to the intervention content or delivery, those mentioned were *lack of Afrikaans speaking Instructors* and *the large size of groups.* Quarterly implementation reports further noted the curricula needed simplification for primary school learners, and an expanded take on sexuality. *Suggestions for intervention expansion* among various stakeholders pointed towards views on current limitations. These included suggestions to work with boys (among schools where girls-only programming occurred), to meet parents, to conduct broader community awareness, to integrate bullying and drug abuse into content, and to extend the program for a full year. Implementation reports confirmed limited parental buy-in served as a barrier.	*NMN seen as professional.* The organization’s professionalism, communication, and flexibility led several stakeholders to further support the program. *Instructors engaging and effective.* A majority of stakeholders described the effectiveness of Instructors in engaging learners through their relatability and passion. *Social Workers viewed as most valuable intervention component.* Most stakeholders spoke of the NMN Social Workers and how they offered a neutral and effective means of support for learners to disclose violence and receive help. *“You see the social worker part is the most effective one its number one because these children appreciate having someone to confide in.”* (Secondary school teacher)
***Outer setting domain***	
*High community crime and poverty* was mentioned by most stakeholders and served as a barrier through several described mechanisms: (1) learners were hungry and distracted, (2) lack of safety for learners and teachers if implemented after school, (3) learners faced much parental abuse and violence, making them more difficult to work with. “*And then with time I was worried because of safety*,* school are being robbed and at the same time I was not able to be here at times*,* so my colleague was the one who was here*,* of which her safety was at stake as well*.” (Secondary school teacher) Implementation reports confirmed that community protests sometimes inhibited implementation, and that safety limited school support for afterschool programming. *Norm of silence around sexual violence.* Several stakeholders described this as dominant among learners and teachers who were uncomfortable with the topic and therefore had trouble reinforcing the curricula, in the case of teachers.	*COVID and increasing community intolerance of sexual violence.* A couple of stakeholders felt that COVID led people to care more about one another and be aware of sexual violence, and one stakeholder described greater community awareness and intolerance of sexual violence, which led them to believe there was good support for the intervention. Implementation reports confirmed a sense that concerns around social ills in the community led to more openness to the intervention.
***Inner setting domain***	
*Resistance among uninvolved teachers inhibited implementation.* The majority of coordinating teachers faced resistance from their colleagues who were not involved in direct communication with the organization. These teachers felt, especially post-COVID, that the intervention interfered with their time with students, and may have also been driven by feeling left out. “*Especially now during COVID and after COVID because my school is still rotating (students)*,* and we don’t see these learners frequently*,* so if going to come during tuition time and say that you want to see the children it becomes very difficult for me even to convince the teachers because they do not see those kids very often*.” (Primary school Head of Department) Implementation reports confirmed the further challenge of conducting the curricula during rotating schedules among learners. They also noted exams as challenging implementation schedules. *Limited time.* Not having enough time to implement a one-hour session was noted by stakeholders and implementation reports. According to reports, content was often not completed and had to overlap with subsequent sessions. “I *still think an hour will suffice and not 40–45 minutes*,* I still feel that’s not enough time.”* (Primary school teacher) “*They sometimes agree to give us one hour for implementing our program*,* but they end up raising the issue of the Department of Education saying that normal curriculum is delivery is 40–45 minutes maximum*,* so why are we not doing the same*.” (Implementation report)	*Good fit with school programming.* More than half of stakeholders described addressing sexual violence as appropriate in the school setting, in-line with Life Skills programming, and as filling a needed gap. *School seen as benefiting from improvements related to the intervention.* Numerous stakeholders reported improvements in: (1) open discussion of sexual violence at school, (2) improved understanding that violence is not normal / wrong, (3) increased disclosure of violence among learners, (4) reduced bullying, (5) improved acceptability of gender and other diversity, (6) cases of sexual violence prevention, and (7) improved confidence of learners. *“I have learners that are shy*,* that don’t even want to lift their hands. I saw them participating you know. That’s how powerful the NMN team was*,* you know*,* to get them to join in.”* (Secondary school teacher) “*Also in their school work there’s so much improvement because they did get a chance to open up and talk about what they feel*,* how they look at things at home*,* and even with the other kids– bullyism*,* and all those sort of things that happen within the school*,* they do get the chance to talk to them about those things and open up and they feel better*.” (Primary school Deputy Principal)
***Individual domain***	
The aforementioned discomfort with the topic sometimes served as a barrier to reinforcing the curricula content.	*Teachers gained knowledge and skills further motivating their support.* One stakeholder in particular described learning to verbally assert herself when she felt a man was violating her space. “*Programs like this make us to be bold*,* also as women…No one is going to say no for you…We also learn things as adults.*” (Primary School Deputy Principal and teacher)

## Discussion

This hybrid type 1 effectiveness-implementation trial, which sought to fill prior gaps in knowledge around the use of an Empowerment-based Self-Defense intervention for sexual violence prevention among adolescent girls in South Africa, provides inconclusive results of intervention effect. While results were not statistically significant, they offer important lessons for future intervention and research on sexual violence prevention. Implementation data points towards challenges in execution of this COVID-adapted curricula and influences outside the program’s scope that should be addressed in future interventions. Simultaneously, qualitative data from stakeholders demonstrated positive impacts in areas that were largely missing from the original intervention logic model and thus omitted from the trial’s quantiative measures. Future trials should consider and incorporate alternative outcomes on the pathway to SV prevention.

Both the small and largely non-significant changes observed in intermediate outcomes, and barriers noted in quarterly reports and by stakeholders, raise questions about the quality of program implementation. Several factors may have contributed to this, including poor retention of program teachings over time since knowledge and attitude measurements were taken several months post-intervention. Monitoring and evaluation data from NMN was used to track fidelity and quality of implementation in the research and non-research schools, as well as pre-post-change in knowledge and attitudes among participants in non-research schools. This data, which, demonstrated high fidelity and quality of implementation in all schools, also found stronger improvements in knowledge and attitudes around self-defense in non-research schools. It is important to note that in non-research schools, post-test surveys are collected immediately after program completion rather than 3-months later and thus capture a shorter recall period. In contrast to prior evaluations of the original intervention that ran for 6 weeks with several refresher sessions and demonstrated reductions in SV [29, 43, 44], this COVID-adapated version was typically implemented within 1–5 weeks with no refreshers. Our results are in-line with a more recently published evaluation of the NMN intervention, which found a non-significant trend towards higher reporting of SV among intervention participants. In addition to being one of the only other evaluations where the NMN program was implemented with primary school students, that evaluation also did not include refresher sessions in intervention implementation [45]. 

Moreover, the trial was conducted in a challenging context during and following the COVID-19 pandemic, by a newly established branch of NMN. Schools were heavily focused on academic recovery, resulting in shortened sessions and limited support from broader school staff. It has also been documented that established risk factors for violence, including structural factors such as unemployment and food insecurity [46–48], were exacerbated during this time and may have diluted the program’s intended impact. School feeding programs can reduce the pressure of economic stress and food insecurity at home, which contribute to violence [49]. Notably these programs did not operate in South Africa while schools were closed during COVID and were found to not sufficiently feed learners, even once schools re-opened [50]. This points toward a need for complementary economic and other outreach to households in times of crisis, which have been shown to serve as cost-effective ways of reducing violence towards adolescents [51]. Future violence prevention programs should consider partnering with community-based or other economic stregthening and feeding programmes such that participants and their families are linked not just with violence response services, but also these critical preventative programmes. Other studies of public health interventions develiered during COVID have similarly documented the challenges that COVID-19 presented to intervention implementation and pointed towards the limits of their adapatations focused almost exclusively on changes in delivery [52, 53].

Another plausible interpretation of the results is that the program increased intervention arm participants’ awareness of violence and comfort in reporting incidents in follow-up surveys. *Knowledge of self-defense* was the only intermediary outcome that was significantly higher in the intervenation arms compared to the control. A shift in comfort in reporting was also described by stakeholders, who highlighted the safe/confidential role played by the NMN Social Workers and the sense of relief gained by students who met with them. This theme was confirmed qualitatively by participants (reported elsewhere), although there was no statistically significant difference in confidence disclosing in this study. Earlier evaluations of the NMN intervention have sometimes observed increased disclosure as a result of intervention participation [44, 54], with or without concurrent increases in the control arms [29]. It is common for research on violence to lead to increased disclosure, especially when addressing gender norms and stigma that contribute to historically low reporting [55]. Greater disclosure can also be seen as a positive step, as it allows women and girls to access the support they need for these experiences. In this study, Social Workers responded to every incident of SV reported through the research surveys, which could have decreased the risk of repeated SV or increased participant’s comfort with disclosure across all arms, diluting our ability to detect a difference across arms.

Although limited evidence suggests that empowerment-based interventions may increase risk of SV for intervention participants due to either overconfidence in using intervention skills or as backlash against their empowerment [56], our findings do not support this. Only 20 participants out of 932 intervention participants reported skill use, defined narrowly as the self-defense skills, suggesting widespread overconfidence and use of self-defense that could increase risk is unlikely. Moreover, qualitative stakeholder reports indicated increased comfort with disclosure of both violence at home and bullying at school, rather than evidence of heightened exposure to violence. Importantly, no intervention participants reported increased risk or adverse events related to skill use in qualitative discussions (reported elsewhere). This suggests that, rather than increasing risk, the intervention may have facilitated safer avenues for disclosure and support-seeking. Additionally, the intervention explicitly addressed safe and strategic skill use, as well as avoidence of risk by first seeking help or escaping a situation of violence, which may have helped mitigate potential backlash effects.

Qualitative feedback from stakeholders also demonstrated positive outcomes beyond the scope of quantitative measurement. These included improvements in participants’ confidence, reduced bullying, and increased acceptance of gender and other forms of diversity. As one stakeholder described of normally shy learners, “*I saw them participating you know. That’s how powerful the NMN team was…to get them to join in*.” Qualitative insights from girls in the intervention also demonstrated enhanced personal safety awareness and assertiveness—not just in relation to SV– but also in interpersonal contexts, such as resisting peer pressure or bullying (reported elsewhere). These findings resonate with other self-defense evaluations, which have consistently described improvements in self-confidence and bodily comfort [13, 57, 58], and may point towards a need to revise how we think about outcomes for future trials. Beyond measuring the direct impact on violence reduction, future studies could explore complementary outcomes, such as improved disclosure, use of a broader range of saftey skills, and self-efficacy and use of consent behaviors within relationships to avoid abandoning potentially useful strategies [59, 60]. These outcomes are influenced by complex social and developmental factors that extend beyond the reach of a single intervention.

The challenges faced during implementation, while not unusual [61], underscore the need to consider program modifications. The importance of adopting a whole-school approach [62]– where all members of the school system are engaged– is one that stakeholders emphasized as crucial for reinforcing program messages. This aligns with existing frameworks on gender- and power-sensitive interventions, which highlight the need to engage both boys and girls, provide developmentally appropriate content, and foster a supportive environment involving teachers, parents, and the community [63]. It is also important given gaps in existing Life Skills curricula content in terms of key topics such as seeking support and reporting SV, the removal of some content which occurred following COVID-lockdowns in order to make up for lost academic time, as well as documented challenges in teacher comfort with these topics [64]. Other modifications such as spreading sessions over a longer period rather than adopting a condensed, rapid delivery, should also be considered. Refresher sessions could support sustained change. Additionally, evidence from other settings has indicated participants’ desire for follow-up sessions and broader community involvement [58]. This further supports the need for sustained engagement beyond the intervention period.

Several limitations must be acknowledged. First, the use of interventionists wearing branded NMN clothing as data collectors presented both benefits and challenges. While their familiarity to participants, particularly intervention participants, may have increased comfort in reporting and was recommended at the time of COVID-19 [65], it could have introduced reporting bias. Although interventionists collected data from different participants than those they directly worked with, their role as facilitators and representatives of the implementing organization may have still influenced responses. Typically, this type of data collection raises concerns about social desirability bias, where participants underreport violence due to the perceived expectations of interventionists delivering a violence prevention program. However, in this case, given that the intervention actively encouraged disclosure and support-seeking, it is also possible that participants in the intervention arm felt more comfortable reporting violence experiences than those in the control arm. This could have led to differential reporting bias, potentially inflating observed intervention effects or obscuring true differences between groups. Additionally, the trial experienced a loss of clusters and a smaller-than-planned sample size, reducing statistical power and our precision of estimated standard errors, particularly for the control arm. This may limit the precision of effect estimates and increase the likelihood of type II errors (failing to detect a true effect). The imbalance in sample sizes between arms also raises concerns about comparability, as differences observed between intervention and control groups may be less reliable. While the analysis adjusted for clustering, the smaller control arm increases variance in effect estimates, potentially making between-group comparisons less stable, however the direction of the results would remain the same. As a result, findings should be interpreted with caution, particularly in relation to effect sizes and statistical significance in subgroup analyses. Another key limitation is that while the intervention of Social Workers in response to survey-based disclosure in both arms was deemed necessary for ethical reasons, it may have biased study results as described above. Lastly, since we evaluated the COVID-adapted model, we cannot draw conclusions about the effectiveness of the “standard” NMN delivery model in South Africa and suggest that future intervention delivery should re-incorporate longer sessions, refreshers, and hands-on pedogological methods.

This study highlights the complexities of implementing ESD programs for adolescent girls in South Africa, especially under the constraints imposed by COVID-19. Although the results were inconclusive and statistically insignificant, they reveal valuable insights about both the challenges and potential benefits of such interventions. The findings emphasize the importance of strong implementation, sustained engagement, and a whole-school approach to create lasting change. However, limitations such as the implementation of a COVID-adapted approach and competing school priorities may have influenced the outcomes, highlighting the need for strategies that ensure deeper integration and continuity. Future studies should measure longer-term change and explore broader outcomes beyond violence reduction, while also accounting for developmental factors and increasing SV risk with age. Ultimately, the program’s qualitative outcomes—improved confidence, reduced bullying, and greater acceptance of diversity—point toward the need to refine how success is conceptualized and measured in future ESD trials.

## Electronic supplementary material

Below is the link to the electronic supplementary material.

Supplementary Material 1

## Data Availability

The raw data supporting the conclusions of this article will be made available by the authors on request.

## Abbreviations

CFIR: Consolidated Framework for Implementation Research
cRCT: Cluster randomized control trial
ESD: Empowerment-based self-defense
IDI: In-depth Interview
LIVES: Listen, Inquire, Validate, Enhance Safety, Support
NMN: No Means No
SV: Sexual violence
WHO: World Health Organization
