# Supplementary material for: Effectiveness of an empowerment-based self-defense program among South African girls: results from a cluster-randomized control trial in schools
Source: BMC Womens Health. 2025 Mar 14;25:119. doi: 10.1186/s12905-025-03647-w (PMC11907951; doi:10.1186/s12905-025-03647-w)
Supplement: Supplementary file 1 — Supplementary Material 1 [file 12905_2025_3647_MOESM1_ESM.docx]

**Additional file 1.**

**Additional table 1. Baseline characteristics of female participants by cluster**

|  | **Girls only arm** | | | | **Girls and Boys arm** | | | | | **Control arm** | | | |  |
| --- | --- | --- | --- | --- | --- | --- | --- | --- | --- | --- | --- | --- | --- | --- |
|  | **1** | **2** | **3** | **4** | **5** | **6** | **7** | **8** | **9** | **10** | **11** | **12** | **13** | **Total** |
| Total | 123 | 84 | 36 | 216 | 118 | 48 | 39 | 99 | 391 | 89 | 90 | 69 | 105 | 1507 |
| School level | Primary | Primary | Primary | Secondary | Primary | Primary | Primary | Secondary | Secondary | Primary | Primary | Secondary | Secondary |  |
| Age - *mean, median (IQR)* | 10.9, 11 (10-11) | 11.0, 11 (10-11.5) | 11.1, 11 11 (10-12) | 14.4, 14 (14-15) | 10.8, 11 (10-11) | 11.0, 11 (10-12) | 11.0, 11 (10-11) | 13.7, 14 (13-14) | 14.8, 15 (14-15) | 11.2, 11 (11-12) | 11.2, 11 (11-12) | 14.6, 14 (14-16) | 15.2, 15 (15-16) | 13.1, 13 (11-15) |
| What do you consider your race/ ethnicity to be? |  |  |  |  |  |  |  |  |  |  |  |  |  |  |
| Black | 120 (98%) | 80 (95%) | 0 (0%) | 209 (97%) | 111 (94%) | 45 (94%) | 2 (5%) | 23 (23%) | 383 (98%) | 81 (91%) | 90 (100%) | 9 (13%) | 103 (98%) | 1256 (83%) |
| Colored | 1 (1%) | 0 (0%) | 34 (94%) | 1 (1%) | 1 (1%) | 0 (0%) | 31 (80%) | 72 (73%) | 0 (0%) | 0 (0%) | 0 (0%) | 34 (94%) | 0 (0%) | 198 (13%) |
| White | 0 (0%) | 1 (1.2%) | 2 (5.6%) | 0 (0%) | 2 (1.7%) | 0 (0%) | 1 (1.6%) | 1 (1%) | 1 (0.3%) | 3 (3.4%) | 0 (0%) | 2 (2.9%) | 0 (0%) | 13 (1%) |
| Household size - mean, median (IQR) | 6.9, 5 (4-8) | 8.0, 6 (5-9) | 7.2, 7 (5-9) | 6.0, 5 (4-7) | 6.6, 6 (4-9) | 7.1, 7 (4-9) | 5.2, 5 (4-6) | 4.6, 5 (4-5) | 5.7, 5 (4-7) | 6.7, 6 (4-8) | 6.7, 5 (4-8) | 5.6, 5 (4-6) | 6.3, 5 (4-8) | 6.2, 5 (4-7) |
| Any food scarcity | 32 (26%) | 27 (35%) | 2 (6%) | 24 (11%) | 26 (23%) | 8 (17%) | 5 (13%) | 13 (13%) | 83 (21%) | 26 (29%) | 14 (16%) | 17 (25%) | 23 (22%) | 314 (21%) |
| Ever had a boyfriend/ girlfriend | 20 (17%) | 27 (34%) | 4 (13%) | 154 (76%) | 26 (23%) | 12 (26%) | 6 (18%) | 52 (59%) | 285 (78%) | 25 (29%) | 19 (21%) | 40 (62%) | 63 (65%) | 733 (52%) |
| Ever had sex | 4 (3%) | 6 (7%) | 0 (0%) | 20 (9%) | 6 (5%) | 1 (2%) | 0 (0%) | 1 (1%) | 57 (15%) | 1 (1%) | 0 (0%) | 0 (0%) | 9 (9%) | 105 (7%) |
| Any SV outcome past 12 months (rape or offline or online harassment) | 20 (16%) | 17 (20%) | 4 (11%) | 83 (38%) | 25 (21%) | 13 (27%) | 3 (8%) | 37 (37%) | 222 (57%) | 16 (18%) | 10 (11%) | 35 (51%) | 43 (41%) | 528 (35%) |
| Any rape past 12 months | 10 (8%) | 10 (12%) | 1 (3%) | 33 (15%) | 11 (9%) | 7 (15%) | 0 (0%) | 12 (12%) | 83 (21%) | 6 (7%) | 6 (7%) | 9 (13%) | 17 (16%) | 205 (14%) |
| Any offline sexual harassment past 12 months | 10 (8%) | 10 (12%) | 4 (11%) | 64 (30%) | 19 (16%) | 9 (19%) | 3 (8%) | 29 (29%) | 190 (49%) | 15 (17%) | 6 (7%) | 31 (45%) | 31 (30%) | 421 (28%) |
| Any online sexual harassment, past 12 months | 4 (3%) | 8 (10%) | 1 (3%) | 47 (22%) | 10 (9%) | 5 (10%) | 1 (3%) | 19 (19%) | 123 (32%) | 10 (11%) | 1 (1%) | 21 (30%) | 22 (21%) | 272 (18%) |

**Additional table 2. Sexual violence outcomes among female participants by cluster**

|  | **Girls only arm** | | | | **Girls and Boys arm** | | | | | **Control arm** | | | |  |
| --- | --- | --- | --- | --- | --- | --- | --- | --- | --- | --- | --- | --- | --- | --- |
|  | **1** | **2** | **3** | **4** | **5** | **6** | **7** | **8** | **9** | **10** | **11** | **12** | **13** | **Total** |
| Total | 120 | 81 | 33 | 173 | 113 | 47 | 37 | 83 | 245 | 86 | 89 | 62 | 81 | 1250 |
| School level | Primary | Primary | Primary | Secondary | Primary | Primary | Primary | Secondary | Secondary | Primary | Primary | Secondary | Secondary |  |
| Any SV outcome past 12 months (rape or offline or online harassment) | 27 (23%) | 18 (22%) | 11 (33%) | 84 (49%) | 27 (24%) | 9 (19%) | 15 (41%) | 30 (36%) | 115 (47%) | 14 (16%) | 8 (9%) | 25 (40%) | 31 (38%) | 414 (33%) |
| Any rape past 12 months | 13 (11%) | 9 (11%) | 3 (9%) | 40 (23%) | 14 (12%) | 4 (9%) | 1 (3%) | 9 (11%) | 41 (17%) | 5 (6%) | 4 (5%) | 8 (13%) | 15 (19%) | 166 (13%) |
| Any offline sexual harassment past 12 months | 16 (13%) | 15 (19%) | 9 (27%) | 59 (34%) | 19 (17%) | 4 (9%) | 13 (35%) | 25 (30%) | 96 (39%) | 8 (9%) | 3 (3%) | 22 (36%) | 25 (31%) | 314 (25%) |
| Any online sexual harassment, past 12 months | 14 (12%) | 10 (12%) | 3 (9%) | 51 (30%) | 13 (12%) | 3 (6%) | 6 (16%) | 13 (16%) | 48 (20%) | 7 (8%) | 2 (2%) | 14 (23%) | 14 (17%) | 198 (16%) |

SV=sexual violence

**Additional table 3. Comparison of sexual violence by intervention versus control at the cluster level**

|  |  | **Intervention** | **Control** | **AdjRR** | **95% CI** | | **p-value** |
| --- | --- | --- | --- | --- | --- | --- | --- |
|  | Primary outcome: sexual violence | 30.9% | 21.8% | 1.34 | 0.99 | 1.79 | 0.05 |
|  |  |  |  |  |  |  |  |
|  | Rape | 10.3% | 8.9% | 1.34 | 0.76 | 2.35 | 0.26 |
|  | Offline sexual harassment | 22.3% | 13.6% | 1.51 | 0.86 | 2.66 | 0.13 |
|  | Online sexual harassment | 13.4% | 9.2% | 1.48 | 0.74 | 2.94 | 0.23 |

Adj RR = adjusted risk ratio; CI = confidence interval. Model adjusted for baseline exposure to sexual violence, and strata defined by school type (primary, secondary) and language (isiXhosa, Afrikaans).

**Additional table 4. Generalized estimating equation model for rape, offline, and online sexual harassment, by arm**

|  |  |  | **Adj RR** | **95% CI** | | | **p-value** |
| --- | --- | --- | --- | --- | --- | --- | --- |
|  | Rape* | Combined intervention arms | 0.83 | 0.50 | 1.39 | | 0.45 |
|  |  | Girls-only | 1.03 | 0.55 | 1.93 | | 0.91 |
|  |  | Girls and boys | 0.73 | 0.38 | 1.42 | | 0.32 |
|  | Offline sexual harassment | Combined intervention arms | 1.36 | 0.92 | 2.04 | | 0.11 |
|  |  | Girls-only | 1.27 | 0.81 | 2.02 | | 0.26 |
|  |  | Girls and boys | 1.29 | 0.84 | 1.95 | | 0.19 |
|  | Online sexual harassment | Combined intervention arms | 1.32 | 0.75 | 2.33 | | 0.29 |
|  |  | Girls-only | 1.88 | 1.09 | 3.24 | | 0.03 |
|  |  | Girls and boys | 1.11 | 0.67 | 1.87 | | 0.63 |
|  |  |  |  |  | |  |  |

Rape was defined as forced, coerced sex, or sex while incapacitated due to drugs or alcohol. Sexual harassment included attempts to obtain a sexual act, unwanted sexual comments or advances, or acts otherwise directed against a person’s sexuality. These were either captured as ‘offline’ events or ‘online’ events that occurred via mobile apps, social networks, texts, or other digital communication. Adj RR = adjusted risk ratio; CI = confidence interval. Fay-Graubard bias-corrected standard errors. All models adjusted for baseline exposure to sexual violence, and strata defined by school type (primary, secondary) and language (isiXhosa, Afrikaans) except the model for *rape** which was not adjusted for strata due to convergence issues.

**Additional table 5. Intermediary outcomes among female participants by visit and arm**

|  | **Girls only arm** | | | | **Girls and Boys arm** | | | | **Control arm** | | | |
| --- | --- | --- | --- | --- | --- | --- | --- | --- | --- | --- | --- | --- |
|  | **Baseline** | **M3** | **M6** | **M12** | **Baseline** | **M3** | **M6** | **M12** | **Baseline** | **M3** | **M6** | **M12** |
| Knowledge and attitudes about self-defense - *mean, median (IQR)* | - | 5.99, 6 (5-7) | - | - | - | 7.52, 8 (6-9) | - | - | - | 5.96, 6 (6-8) | - | - |
| Gender norms and attitudes - *mean, median (IQR)* | 22.61, 23 (20-25) | - | 21.91, 22 (20-24) | 22.02, 22 (20-24) | 22.68, 23 (21-24) | - | 22.48, 23 (21-25) | 23.15, 23 (21-25) | 22.22, 22 (20-24) | - | 21.99, 22 (20-24) | 22.34, 23 (21-24) |
| Victim blaming attitudes - *mean, median (IQR)* | 11.71, 12 (10-13.5) | - | 12.09, 12 (10-14) | 12.25, 12 (10-14) | 12.39, 13 (11-14) | - | 12.94, 13 (11-15) | 13.06, 13 (11-15) | 11.75, 12 (10-14) | - | 12.25, 12 (11-14) | 12.23, 12 (10-14) |
| Confidence reporting SV in the future | 284 (73) | - | 248 (75) | 213 (77) | 284 (67) |  | 379 (83) | 351 (80) | 248 (73) |  | 227 (77) | 213 (77) |
| Use of intervention behaviors | - | - | 11 (3%) | 0 (0%) | - | - | 9 (2%) | 0 (0%) | - | - | 2 (1%) | 0 (0%) |

IQR=interquartile range; M=month.

**Additional table 6. Generalized estimating equation model for secondary outcomes, comparing intervention to control**

|  |  | **Intervention effect (coefficient)** | **95% CI** | | **p-value** |
| --- | --- | --- | --- | --- | --- |
|  | Knowledge and attitudes about self-defense | 0.42 | 0.16 | 0.68 | 0.007 |
|  | Gender norms and attitudes | -0.03 | -0.25 | 0.21 | 0.79 |
|  | Victim blaming attitudes | 0.17 | -0.10 | 0.45 | 0.18 |
|  | Change in confidence reporting SV in the future | 0.10 | -0.12 | 0.32 | 0.33 |
|  |  |  |  |  |  |

CI = confidence interval. Fay-Graubard bias-corrected standard errors. All models adjusted for baseline exposure to sexual violence, and strata defined by school type (primary, secondary) and language (isiXhosa, Afrikaans). Models were also adjusted for their own baseline values except *knowledge* which was not measured at baseline and *change in confidence reporting SV*, which incorporated baseline values into its measurement.

**Additional table 7. Generalized estimating equation model for secondary outcomes, by arm**

|  |  |  | **Intervention effect (coefficient)** | **95% CI** | | **p-value** |
| --- | --- | --- | --- | --- | --- | --- |
|  | Knowledge and attitudes about self-defense | Girls-only | 0.17 | -0.08 | 0.41 | 0.15 |
|  |  | Girls and boys | 0.56 | 0.35 | 0.76 | 0.001 |
|  | Gender norms and attitudes | Girls-only | -0.16 | -0.57 | 0.25 | 0.38 |
|  |  | Girls and boys | 0.08 | -0.18 | 0.33 | 0.18 |
|  | Victim blaming attitudes | Girls-only | 0.06 | -0.29 | 0.40 | 0.71 |
|  |  | Girls and boys | 0.27 | -0.02 | 0.56 | 0.06 |
|  | Change in confidence reporting SV in the future | Girls-only | 0.09 | -0.21 | 0.39 | 0.52 |
|  |  | Girls and boys | 0.11 | -0.12 | 0.34 | 0.30 |
|  |  |  |  |  |  |  |

CI = confidence interval. Fay-Graubard bias-corrected standard errors. All models adjusted for baseline exposure to sexual violence, and strata defined by school type (primary, secondary) and language (isiXhosa, Afrikaans). Models were also adjusted for their own baseline values except *knowledge* which was not measured at baseline and *change in confidence reporting SV*, which incorporated baseline values into its measurement.
